# A new fossil plesiomorphic flat bug (Aradidae) suggests widespread flower visiting in Heteroptera during the Mesozoic

**DOI:** 10.1038/s41598-025-15559-8

**Published:** 2025-08-19

**Authors:** Péter Kóbor, Márton Szabó

**Affiliations:** 1 Department of Zoology, HUN-REN Centre for Agricultural Research Plant Protection Institute, Martonvásár, 2462 Hungary; 2 Department of Palaeontology and Geology, Hungarian National Museum Public Collection Centre – Hungarian Natural History Museum, Budapest, 1082 Hungary; 3 Department of Palaeontology, ELTE Eötvös Loránd University, Institute of Geography and Earth Sciences, Budapest, 1117 Hungary

**Keywords:** Heteroptera, Aradidae, Fossil, Iridescence, Anthophily, Palaeontology, Entomology, Palaeontology, Taxonomy, Palaeoecology

## Abstract

The phenomenon of flower visiting (anthophily) and the pollination, though becoming prevalent with the rise of flowering angiosperms, hypothesized to have originated from the antagonistic relationship of florivory between insects and gymnosperms in the Upper Jurassic. Though not commonly known, this behaviour has been documented in several instances among the representatives of the suborder Heteroptera, i.e., the true bugs. Here, we describe *Shaykayatcoris michalskii* gen. nov., sp. nov., the first known representative of the plesiomorphic flat bug (Aradidae) subfamily Prosympiestinae in the Upper Cretaceous Burma Terrane amber fauna (Lowermost Cenomanian, ca. 99 Mya). Besides expanding the knowledge on the heteropteran insect fauna of the Lagerstätte, the discovery of this new true bug provides intriguing insights into the evolutionary history of true bugs. First, the new record corroborates that the subfamily Prosympiestinae is a Gondwanan relict group among the representatives of flat bugs. Second, this insect presents the first known incidence of iridescent colouration in flat bugs with a tentative role of camouflage, suggesting a more exposed lifestyle compared to the apomorphic lineages of flat bugs, which have adapted to live under tree bark. Third, the iridescence and the high amount of pollen among the syninclusions suggest that the insect was likely to be anthophilous. The suspected presence of anthophily in such a specialised heteropteran insect group like Aradidae suggests that anthophily was more widespread among the Mesosoic true bugs than it can be observed in the case of extant taxa.

**Supplementary Information:**

The online version contains supplementary material available at 10.1038/s41598-025-15559-8.

## Introduction

The intense diversification of flowering terrestrial angiosperms, also known as the Angiosperm Terrestrial Revolution (abbreviated as ATR, ca. 100–50 Mya), resulted in the formation of a plethora of ecological niches to be occupied^1^. This event played a dual role in the development of insect biodiversity, mitigating the extinction of specific lineages while promoting the rise and radiation of others^2^. The most important of these ecological roles was pollination, which evolved in multiple insect orders, e.g., beetles, hymenopterans, or true bugs, first occurring during the Upper Jurassic and originally associated with gymnosperms, but becoming more prevalent with the diversification of flowering plant lineages, and its origin is suspected to lie in the antagonistic insect-plant relationship, i.e., florivory^3–5^. Though not a group commonly known as pollinators, the behaviour of antophily and the associated pollination is a well-documented phenomenon in multiple families of the suborder Heteroptera, e.g., plant bugs (Miridae), seed bugs (Lygaeidae), or stink bugs (Pentatomidae)^6–11^. Even zoophytophagous, i.e., predators alternatively utilizing plant-based food, are visiting flowers to feed on the pollen or nectar^12–14^.

The family Aradidae, commonly known as the flat bugs, is a highly specialized group of the infraorder Pentatomomorpha in terms of morphology and lifestyle^15,16^. Their body is flattened dorsoventrally in most of the included species, and stylets of the piercing-sucking mouthpart are extremely elongated, situated coiled in the head capsule in a resting position. These peculiarities are adaptations to their unique lifestyle, i.e., living under tree bark and feeding on the fungi growing there^17^. An exception to this is the plesiomorphic subfamily Prosympiestinae Usinger and Matsuda, 1959; these aradid bugs are not flattened in habitus and live in detritus or under logs lying on the ground, not exclusively under tree bark^18^. Representatives of Prosympiestinae are distributed predominantly in eastern Australia and New Zealand, with the single genus of tribe Llaimacorini Kormilev, 1964 known from Chile. This distribution pattern suggests a Gondwanan origin for the group^15,18,19^.

Flat bugs have a fossil record dating back to the Early Cretaceous with the earliest records from the Lagerstätten of western Mongolia and Russia^16^. The family is represented by 19 species in the diverse and intriguing fauna of the Late Cretaceous Burmese amber (Lowermost Cenomanian, ca. 99 Mya)^20–22^ It is to be noted that these species are related to apomorphic lineages, e.g., Aradinae or Mezirinae, and display an amalgamation of characteristics of various genera^21,23^. On the other hand, to date, more ancient lineages, e.g., Prosympiestinae or Isoderminae, lack records from Burmese amber.

Here, we report the first known representative of the plesiomorphic subfamily Prosympiestinae from Burmese amber and discuss its biogeographical and evolutionary ecological implications with an emphasis on the iridescent colouration and a suspected anthophilous lifestyle for this bug.

## Results

### Systematic palaeontology

Order **Hemiptera** Linnaeus, 1758.

Suborder **Heteroptera** Latreille, 1810.

Infraorder **Pentatomomorpha** Leston, Pendergrast and Southwood, 1954.

Superfamily **Aradoidea** Brullé, 1836.

Family **Aradidae** Brullé, 1836.

Subfamily **Prosympiestinae** Usinger and Matsuda, 1959.

***Shaykayatcoris*** Kóbor and Szabó gen. nov.

**Fig. 1:**
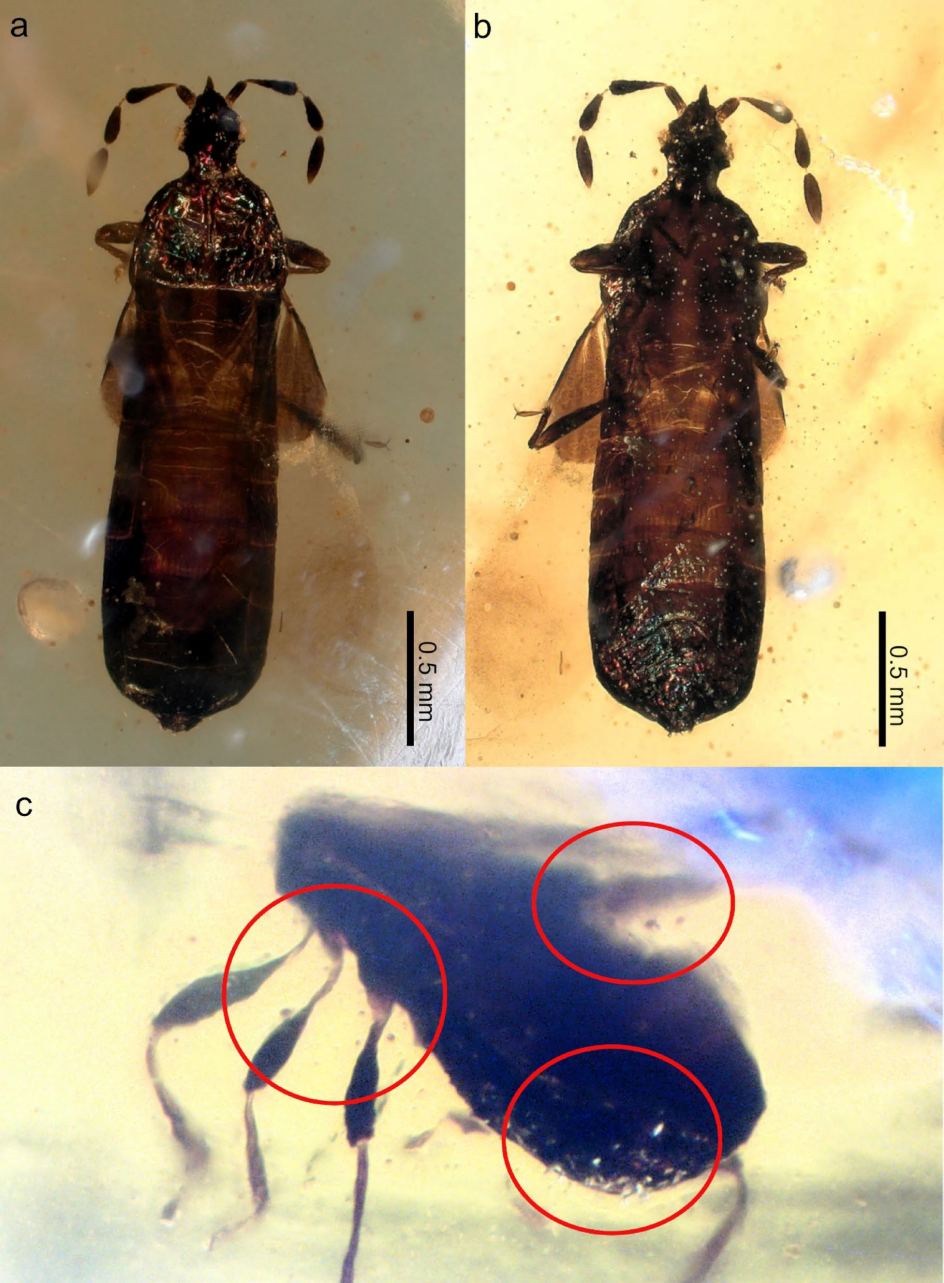
*Shaykayatcoris michalskii* gen. nov, sp. nov.: (**a**) dorsal habitus, (**b**) ventral view, (**c**) pollen grains attached to integument (marked with red circle).

**Fig. 2:**
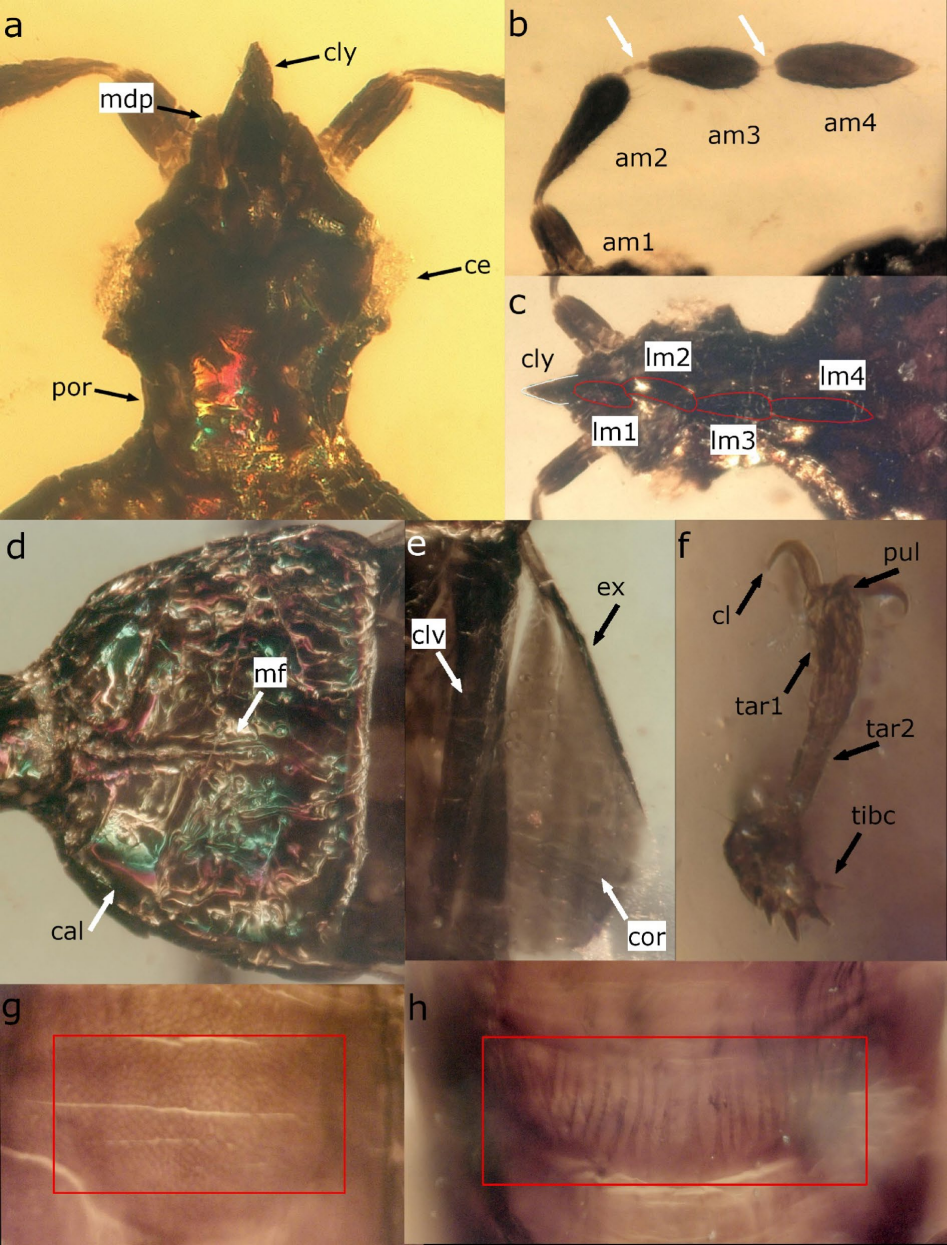
Morphological characters of *Shaykayatcoris michalskii*: (**a**) head in dorsal view (lettering: ce – compound eye, cly – clypeus, mdp – mandibular plate, por – postocular region), (**b**) antenna (lettering: am1 – scapus, am2 – pedicel, am3 – basiflagellomere, am4 – distiflagellomere; pre- and intraflagelloid indicated with arrows), (**c**) head in ventral view (lettering: cly – clypeus, lm I–IV: labiomeres; apex of clypeus outlined with white, antennomeres outlined with red), (**d**) pronotum (lettering: cal – pronotal callosities, mf – median furrow of pronotum), (**e**) hemelytron without membrane (lettering: clv – clavus, cor – corium, ex – exocorium), (**f**) apex of tibia and tarsus with appendages (lettering: cl – tarsal claw, pul – pulvilli, tar1-2 – tarsomere I–II, tibc – tibial comb), (**g**) integument of abdominal tegite III (corrugate area indicated with red), h. stridulitrum on abdominal venter (indicated with red) (all images are not to scale).

**Fig. 3:**
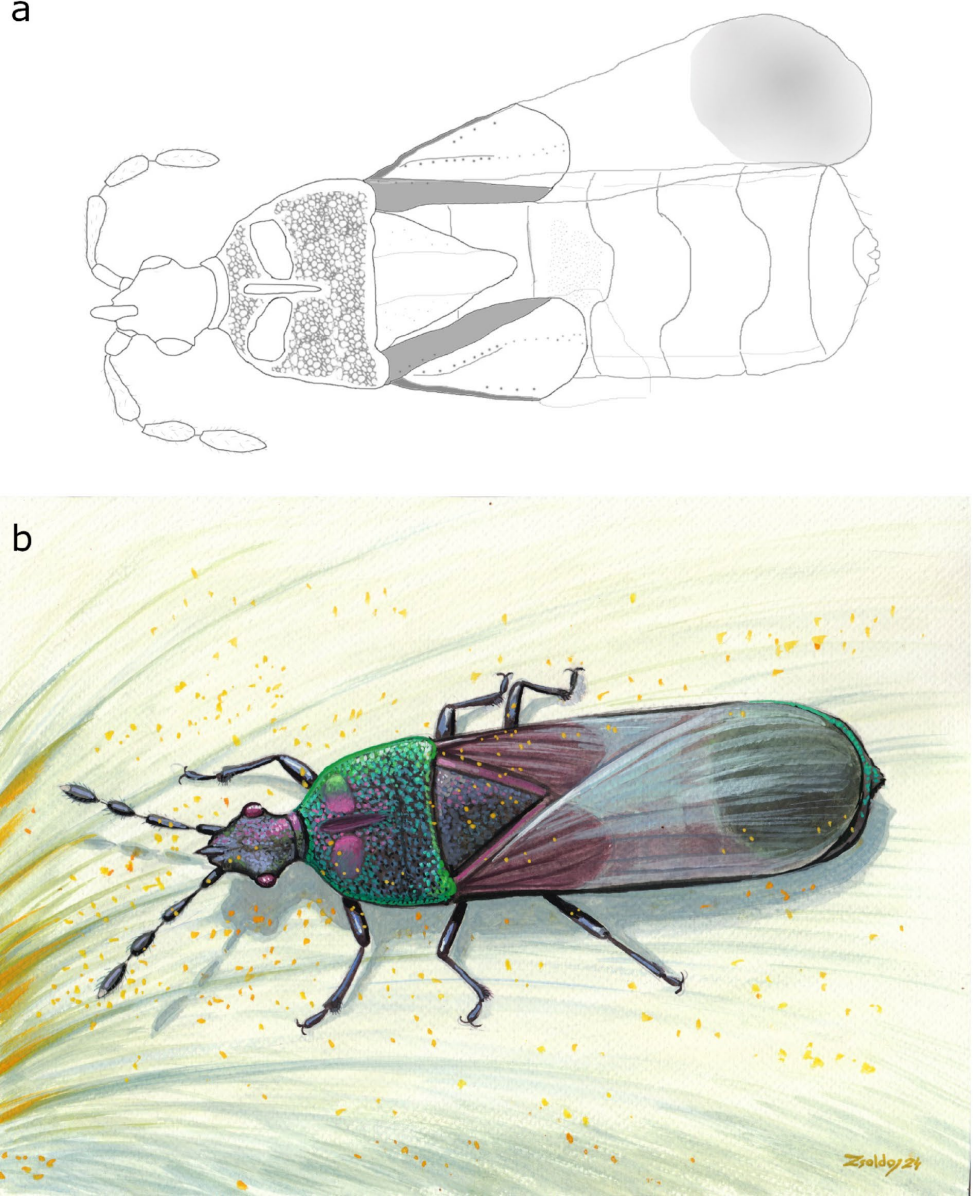
Reconstructions of *Shaykayatcoris michalskii* gen. et sp. nov.: (**a**) line drawing of the second author (parts of femora visible in dorsal view were omitted), (**b**) artistic reconstruction by Márton Zsoldos, Hungarian palaeoartist.

**Fig. 4:**
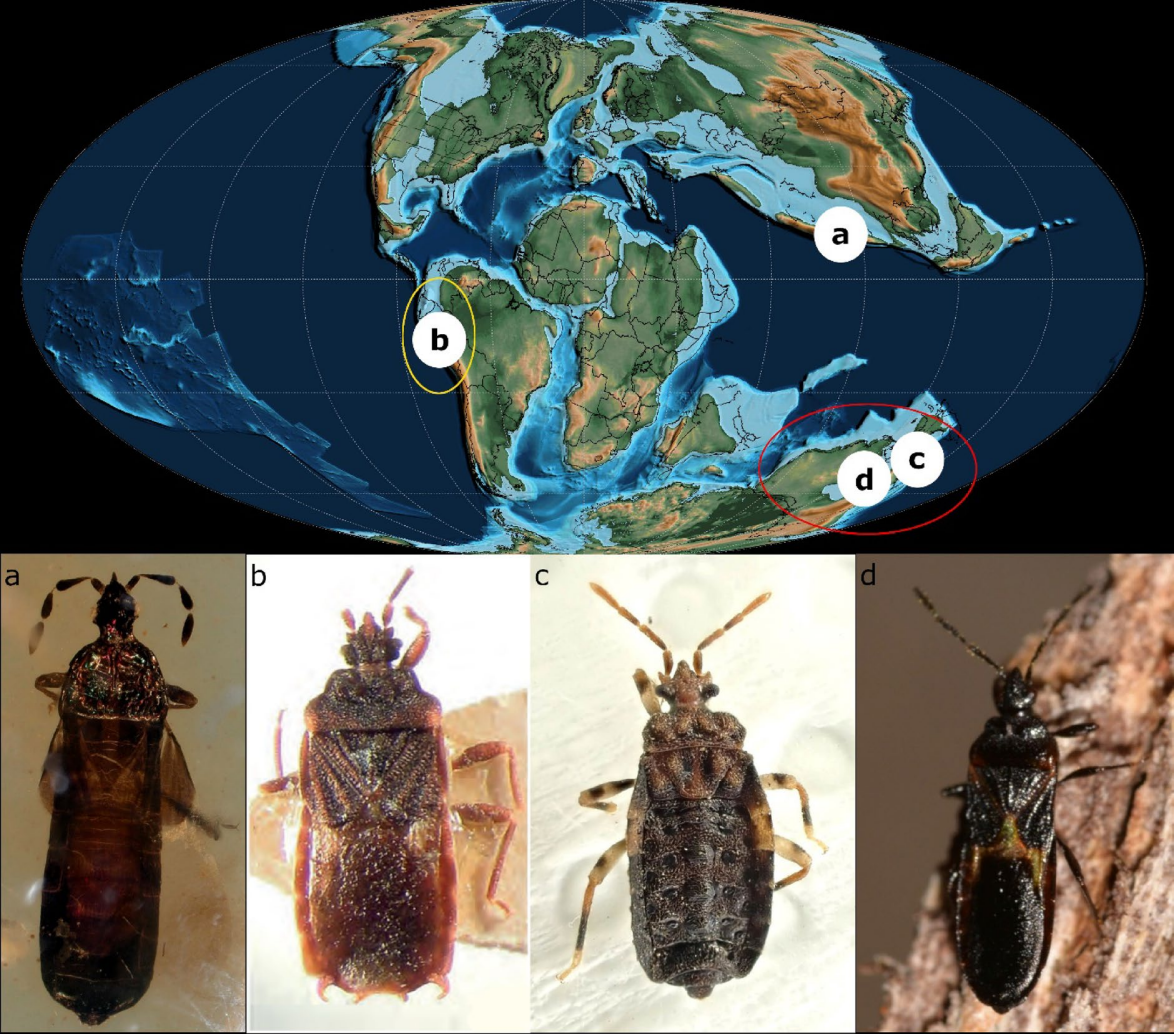
Distribution of subfamily Prosympiestinae projected to topographic map of the Upper Cretaceous period (Albian, 105 Mya; map source Scotese et al. 2025 under CC BY 4.0 license): (**a**) *Shaykayatcoris michalsikii* gen. nov.,sp. nov., (**b**) *Llaimacoris penai* Kormilev, 1964 (photo republished with modification: Heiss, 2017 Fig. 16), (**c**) *Adenocoris* sp. (photo: Grey Smith, iNaturalist.org, license: CC BY 4.0), (**d**) *Prosympiestus* sp. (photo: Nick Lambert, iNaturalist.org, license: CC BY-NC-SA 4.0) (lettering on map corresponds to that on habitus photos; red circle indicates the area of Prosympiestini Usinger & Matsuda, 1959, yellow circle indicates the estimated area of Llaimacorini Froeschner & Kormilev, 1987).

LSID: http://zoobank.org:pub:B7B8EEA4-1277-4AE6-A9EE-2F1AC11F2BC6.

(Figs. 1, 2, 3 and 4b)

Type species: *Shaykayatcoris michalskii* sp. nov., by present designation.

#### Description

Habitus elongate, sub-cylindrical, with dorsum slightly flattened (Figs. 1a and c and 3a). Integument predominantly lacking pubescence (indicated where present) (Fig. 1a, b). Head (Fig. 2a–c) slightly elongate, deltoidal; vertex integument appears smooth, lacking observable punctures or corrugation (Fig. 2a). Head with conspicuous postocular region (por) (Fig. 2a). Compound eyes circular, ocelli missing. Clypeus (cly) well-defined, lateral margins constricted basally, apex pointed. Mandibular plates (mdp) developed, reaching up to approximately half of the clypeus length. Antenniferous tubercles inconspicuous, antennae inserted anteriorly. Scapus (am1) cylindrical; pedicel (am2) and basiflagellomere (am3) club-shaped; distiflagellomere graniform (am4) (Fig. 2b). Pedicel and flagellomeres with relatively dense semi-erect setae. Pre- and intraflagelloid present between pedicel and flagellomeres, thread-like. Rostrum attached well behind the apex of the clypeus (Fig. 2c). Labiomere I–III (lm1–3) appear to be subequal in length, labiomere IV (lm4) longest. Apex of labium reaches up to the anterior margin of the prothorax approximately (hard to assess due to slight distortion). Gular area smooth. Thorax. Pronotum (Fig. 2d) semicircular with a narrow, slightly distinct collar; lateral margins rounded; posterior margin slightly concave. Pronotal callosities well-defined, ovoid, separated by a median longitudinal furrow reaching up to two-thirds of the pronotum length. Integument of pronotum with dense, deep punctuation. Scutellum triangular, with apex pointed (Fig. 1a). Integument of scutellum with fine punctuation. Hemelytron tripartite with clavus, corium, and membrane discernible (Figs. 1a and 2e). Clavus (clv) narrow with margins subparallel; claval commissure present, but minute. Corium (cor) relatively weakly sclerotized with posterior margin convex, rounded, and exocorium (ex) thickened. Membrane well-developed, nearly twice as long as corium, lacking venation. Hind wings unobservable. Thoracic pleurites and sternites finely punctate. Peritreme auricular situated somewhat anteriorly to the metacoxa. Coxal cavities separated conspicuously by thoracic sternites, open posteriorly. Femora (Fig. 1b) club-shaped, fore femora slightly incrassate. Tibiae (Fig. 1b) thin, cylindrical, slightly thickened apically, with a tibial comb at apex (Fig. 2f, tibc). Tarsi (Fig. 2f) are two-segmented, with tarsomere I (tar1) slightly longer than tarsomere II (tar2). Claws slender, evenly curved. Unguitractor plate bearing a pair of spatulate pulvilli and a pair of small setiform parempodia. Abomen. Abdominal tergite III with corrugate patch medially (Fig. 2g). Sutures between tergites IV-V-VI curved medially, scent gland cars visible (Fig. 1a). Abdominal sternite III–V with wrinkled stridulitrum (Fig. 2h). Abdominal sternites VI-VII with sparse, short pubescence (Fig. 1b, c). Ovipositor short, not extending beyond the genital segment.

#### Differential diagnosis

*Shaykayatcoris* gen. nov. possesses the following synapomorphies shared with representatives of the tribe Prosympiestini: gular area smooth (in Llaimacorini gular area with a shallow longitudinal groove), peritreme situated anteriorly to hind coxa (in Llaimacorini peritreme situated close to the middle coxa). The bristle of the peritreme characteristic of tribe Prosympiestini is not observable, but it may have been broken off when trapped in resin. *Shaykayatcoris* gen. nov. is macropterous, possessing a triangular scutellum (shared synapomorphy with *Prosympiestus* Bergroth, 1894; other prosympiestine genera are brachypterous), length of head greater than the width (shared synapomorphy with *Prosympiestus*, length of head distinctly shorter than width in the other prosympiestine genera), antenniferous tubercle reduced (autapomorphy, antenniferous tubercles well-developed or even produced laterally in other prosympiestine genera), postocular region slightly concave (autapomorphy, postocular region convex or straight in other prosympiestine genera), width of pronotum is 1.3× greater than length (width of pronotum is at least twice greater than length in other prosympiestine genera), scutellum is slightly shorter than pronotum (autapomorphy, scutellum is at least somewhat shorter in other prosympiestine genera), hemelytron with fine, sparse punctuation at claval furrow and along veins of corium (autapomorphy, hemelytron extensively and strongly punctate in *Prosympiestus*).

#### Etymology

Generic name masculine. The name of the genus is derived from the Burmese word  (shayyhkayat), meaning ’ancient’ and the Greek κοριός (koriós = coris), meaning ’bug’.

***Shaykayatcoris michalskii*** Kóbor and Szabó sp. nov.

LSID: http://zoobank.org:act:3206A9D0-38BE-48FF-8AE4-9E86B6BF9AB7.

**Material examined. Holotype**, female: The holotype is deposited in the Palaeontological Collection of the Naturhistorisches Museum, Vienna, Austria, under the curatorial number 2025/0175/0001. The specimen is included in a transparent, yellow amber piece with dimensions ca. 12.2 × 6.1 × 4.0 mm, with multiple syninclusions (plant debris and unidentified insect). The amber piece contains large amounts of pollen from which several grains are attached to the body of the specimen (Fig. 2c).

#### Type locality and horizon

Upper Cretaceous, lower Cenomanian (98.79 ± 0.62 Ma, according to Shi et al.^24^; from an amber mine in Hukawng Valley, Tanai Township, Myitkyina District, Kachin State, northern Myanmar.

#### Description

##### Colouration

The base colour of the insect appears to be dark fuscous with antennae, clavus, and exocorium darker in hue variably (to almost blackish). Integument iridescent, colour predominantly green, sometimes infused with purple (Figs. 1a and 3b). Distinct pattern not recognisable. The membrane of hemelytron is hyaline with a large, rounded infuscate spot apically. Base of femora ochraceous.

##### Structure

Monospecific genus. As in the generic description.

##### Measurements (in mm)

Total length of body: 2.48; head length: 0.35, width: 0.28; antennomeres I-IV (excluding pre- and intraflagelloid): 0.08, 0.20, 0.14, 0.18; pronotum length: 0.44, - width: 0.59; scutellum length: 0.42, width: 0.35; corium length: 0.59; length of hemelytron: 1.45; tarsomere I-II: 0.06, 0.04.

##### Differential diagnosis

*S*. *michalskii* is the single known representative of the genus so far.

#### Etymology

The species is named after Artur Michalski, a Polish amber collector (predominantly specialized in Baltic amber) who donated the specimen to the authors for scientific purposes. With the naming, authors intend to encourage collectors and traders to cooperate with scientists to reveal the palaeobiodiversity trapped in amber.

## Discussion

The studied insect possesses four-segmented antennae with a fusiform distiflagellomere; four-segmented labium with all labiomeres well-developed; hemelytron tripartite, i.e., clavus, corium, and membrane distinct. These characteristics meet the circumscription of Pentatomomorpha^15^. The familial placement in Aradidae is supported by the straight antennae, the presence of compound eyes, the absence of ocelli, two-segmented tarsi, and the lack of abdominal trichobothria^15,25^. *Shaykayatcoris* gen. nov. possesses the following combination of characters: habitus subcylindrical, mandibular plates well-developed, rostrum arising well beyond the apex of the clypeus, hemelytron without “line of weakness”, corium with a single submarginal vein, peritreme well-developed. This combination of characters meets the definition of the Gondwanan relict group, Prosympiestinae Usinger & Matsuda, 1959. The tribal classification of Prosympiestinae is based on the groove of the gular area and the characteristics of the peritreme^26^. In the studied insect, the gular area is completely smooth, lacking groove-like structures. The peritreme is situated near the hind coxa and appears to be oval, though it lacks bristle-like setae. These characteristics suggest the placement of the new genus in the tribe Prosympiestini Usinger & Matsuda, 1959. Within Prosympiestini, the generic assignment is determined by e.g., the shape of the antenniferous tubercle, the arrangement of the scutellum, and the placement of the spiracles. The spiracles are not visible on the studied specimen, but the antenniferous tubercles appear to be reduced, inconspicuous, which is an autapomorphy regarding the tribe. The scutellum is triangular with the apex pointed; this character is shared with the genus *Prosympiestus*. Further autoapomorphies, e.g., concave postocular region, ratio of pronotum length to width, or punctuation of hemelytron, support the uniqueness of this new true bug. Thus, we establish a new genus, *Shaykayatcoris* gen. nov., to accommodate this species.

The representatives of Prosympiestinae are distributed in Eastern Australia, New Zealand, and Chile, which distribution pattern suggests a Gondwanan origin for this plesiomorphic group (Fig. 4b–d)^15,18,26^. The fossil of *Shaykayatcoris* was found in Burmese amber, which was formed in the Burma Terrane (Fig. 4a). This landmass separated from Gondwana after the Late Triassic; hence, its biota originated from Gondwanan ancestors but evolved in isolation for several million years^27,28^. Considering these, the presence of a prosympiestine flat bug in the Burma Terrane amber fauna supports the Gondwanan origin of this flat bug subfamily and underpins the ties of the whole fauna too.

One of the most striking features of *Shaykayatcoris michalskii* is the predominantly iridescent integument (Figs. 1a and b and 3b). Iridescence is a form of structural colouration which is widespread in several insect orders, e.g., cockroaches, hymenopterans, or coleopterans^29–32^. In true bugs, the phenomenon has been observed in various taxa, e.g., assassin bugs (family Reduviidae)^33,34^ or stink bugs and allies (superfamily Pentatomoidea)^35–37^. In Pentatomoidea, a partly iridescent fossil representative, *Edessa protera* Poinar & Thomas, 2012, is also known^38^. However, in the case of flat bugs, which adapted mostly to live under tree bark, thus benefiting little from the advantages meant by iridescent colouration (as described below), *Shaykayatcoris michalskii* gen. nov., sp. nov. is the first known representative to display this characteristic.

Considering the suspected ecological function of the iridescent colouration, two scenarios are recorded in extant insects: aposematism or camouflage. In the case of aposematisms, i.e., warning colouration, the iridescence is mostly combined with vivid colours and complex patterns^39,40^. However, none of these are observed in *Shaykayatcoris* gen. nov. Iridescence in insects was also interpreted as a form of camouflage, increasing the chance of survival for iridescent prey animals against visually hunting predators^41,42^. This case is corroborated by the size and the tentative ecological niche of the insect: this small bug is preserved together with several botanical syninclusions, such as a large amount of pollen with multiple grains attached to the body (Fig. 1c), suggesting a suspected anthophilous-pollinator lifestyle^4,43^. Numerous important pollinator insect taxa are known to have iridescent or metallic colouration, which may be linked to their visually exposed life-style^44–46^.

Here, we described *Shaykayatcoris michalskii* gen. nov., sp. nov., the first known representative of the plesiomorphic flat bug subfamily Prosympiestinae from Upper Cretaceous Burmese amber. The discovery of the species is not only the first reported occurrence of the group in the Burma Terrane amber fauna, but also has biogeographic and ecological implications. The presence of Prosympiestinae in Burmese amber supports the assumption that this plesiomorphic subfamily is one of the Gondwanan relict groups of heteropteran insects. The iridescent colouration of the insect suggests an exposed lifestyle, which is unusual among the representatives of Aradidae, a family that has completely adapted to living under tree bark. However, it must be noted that extant prosympiestine flat bugs are not exclusively found under tree bark, but predominantly live in plant detritus. Considering that Prosympiestinae is plesiomorphic regarding Aradidae, it is assumed that the ancestors of flat bugs, at least partly, transitioned from geophilous-arboreal habitat use through detritus-dwelling to under the tree bark. The phenomenon of iridescence paired with the large amount of pollen found in the amber piece and stuck to the body surface suggests an antophilous-pollinator lifestyle. Though the grains appear to be deformed, the identification of the plant is not possible. The mere incidence of the phenomenon puts anthophily in Heteroptera in a new perspective. The phenomenon, though, in several cases well-documented, is relatively rare in Heteroptera, only present in 9 of the 104 extant families of all hemipteran insects, i.e., true bugs, cicadas, and allies^4^. The most well-known cases of pollination are recorded from plant bugs^5,9^, in other groups, it is considered incidental. However, this new record suggests that this was different earlier in the evolutionary history of these insects, and anthophily may have been more widespread in the distant past. The hypotheses regarding the transition in habitat use and the relegated anthophily are subject to further investigation, preferably in a phylogenetic context. However, the prerequisite for this is the accumulation of additional fossil evidence that can be obtained through the intensive exploration of the Burma Terrane amber fauna.

## Methods

The burmite piece containing the specimen was donated to the first author by Artur Michalski. According to our knowledge, the amber piece was mined and sourced before May 2017. The genuineness of the amber piece was assessed with the methods described by Poinar^47^, and all three tests proved that the amber piece is original. The amber piece did not go under further processing, i.e., cutting or extensive polishing; only slight polishing was applied to remove fine scratches before photographing. Observations and photodocumentation were performed with the use of a Kern Optics OZL 466 stereoscopic microscope mounted with a Kern Optics OCD 832 (5 MPix) microscope camera (operating software: Kern & Sohn Microscope VIS 2.0 Pro) and Keyence VHX5000 digital microscopes, using transmitted and incident lighting simultaneously. Images were processed and plates were arranged using GIMP 2.10.38 image manipulation software. For Fig. 4, the map of Scotese et al.^48^ was used to interpret the distribution. Morphological terminology was adapted from Tsai et al.^49^, Usinger & Matsuda^25^, and Lariviére & Larochelle^18^. Nomenclatural references are provided in Supplementary Data S1. The tentative ecological role of the insect was determined following the methodology of Peña-Kairath et al.^4^.

## Supplementary Information

Below is the link to the electronic supplementary material.

Supplementary Material 1

## Data Availability

The datasets used and analysed during the current study are included in this published article. The holotype is deposited in the Palaeontological Collection of the Naturhistorisches Museum, Vienna, Austria, under the curatorial number 2025/0175/0001.
