# Supplementary material for: A new fossil plesiomorphic flat bug (Aradidae) suggests widespread flower visiting in Heteroptera during the Mesozoic
Source: Sci Rep. 2025 Aug 19;15:30282. doi: 10.1038/s41598-025-15559-8 (PMC12365054; doi:10.1038/s41598-025-15559-8)
Supplement: Supplementary file 1 — Supplementary Material 1 [file 41598_2025_15559_MOESM1_ESM.docx]

**Supplementary Data S1 – Nomenclatural references**

Prosympiestinae Usinger and Matsuda, 1959 – Usinger, R. L., & Matsuda, R. *Classification of the Aradidae (Hemiptera-Heteroptera)*. (British Museum, London, 1959).

Llaimacorini Kormilev, 1964 – Kormilev N. A. 1964. Neotropical Aradidae XIII (Heteroptera: Aradidae). *J. N. Y. Ent. Soc.* 72: 112–119 (1964).

Hemiptera Linnaeus, 1758 – Linnaeus, C. *Systema Naturae* (10th ed.). (Lackington & Allen, London, 1758).

Heteroptera Latreille, 1810 – Latreille, P. A. *Considérations générales sur l'ordre natural des animaux composant les classes des Crustacés, des Arachnides et des Insectes; avec un tableau méthodique de leurs genres, disposés en familles*. (F. Schoell, Paris, 1810).

Pentatomomorpha Leston, Pendergrast and Southwood, 1954 – Leston, D., Pendergrast, J. G., & Southwood, T. R. E. Classification of the terrestrial Heteroptera (Geocorisae). Nature, 174(4419), 91-92 (1954).

Aradoidea / Aradidae Brullé, 1836 – Brullé, A. *Histoire naturelle des insectes, traitant de leur organisation et de leurs mœurs en général, et comprenant leur classification et la description des espèces. Orthoptères et Hémiptères*, IX(2 parts), (Pillot, Paris, 1836).

Prosympiestus Bergroth, 1894 – Bergroth, E. Fortsatta bidrag till Acadidernas kannedom. *Ent. Tidsk*. 15: 97–118 (1894).
